# A smart devices based secondary prevention program for cerebrovascular disease patients

**DOI:** 10.3389/fneur.2023.1176744

**Published:** 2023-06-02

**Authors:** Francesco Motolese, Fioravante Capone, Alessandro Magliozzi, Carlo Vico, Gianmarco Iaccarino, Emma Falato, Fabio Pilato, Vincenzo Di Lazzaro

**Affiliations:** ^1^Department of Medicine and Surgery, Unit of Neurology, Neurophysiology, Neurobiology and Psichiatry, Università Campus Bio-Medico di Roma, Roma, Italy; ^2^Fondazione Policlinico Universitario Campus Bio-Medico, Roma, Italy

**Keywords:** stroke, cerebrovascular disease, prevention, atrial fibrillation, smart devices, mHealth

## Abstract

**Background:**

Commercially available health devices are gaining momentum and represent a great opportunity for monitoring patients for prolonged periods. This study aimed at testing the feasibility of a smart device-based secondary prevention program in a cohort of patients with cryptogenic stroke.

**Methods:**

In this proof-of-principle study, patients with non-disabling ischemic stroke and transient ischemic attacks (TIA) in the subacute phase were provided with a smartwatch and smart devices to monitor several parameters – i.e., oxygen saturation, blood pressure, steps a day, heart rate and heart rate variability - for a 4-week period (watch group). This group was compared with a standard-of-care group. Our primary endpoint was the compliance with the use of smart devices that was evaluated as the number of measures performed during the observation period.

**Results:**

In total, 161 patients were recruited, 87 in the WATCH group and 74 in the control group. In the WATCH group, more than 90% of patients recorded the ECG at least once a day. In total, 5,335 ECGs were recorded during the study. The median blood pressure value was 132/78 mmHg and the median oxygen saturation value was 97%. From a clinical standpoint, although not statistically significant, nine atrial fibrillation episodes (10.3%) in the WATCH group vs. 3 (4%) in the control group were detected.

**Conclusion:**

Our study suggests that prevention programs for cerebrovascular disease may benefit from the implementation of new technologies.

## Introduction

Stroke is the major contributor to disability-adjusted life years (DALY) and the leading cause of death among neurological disorders (1). The prevalence of cerebrovascular disease is expected to increase in the next decades because of ageing population (2), while the stroke-related mortality has decreased considerably owing to the improvement of acute phase management (2). Antithrombotic therapies and adequate prevention strategies represent the mainstay of treatment to reduce the risk of stroke recurrence.

Despite recent advances in secondary prevention, the rate of stroke recurrence at 5 years ranges from 10 to 20% of patients according to different studies, and it has not substantially changed in the last decade (3, 4). Besides, some stroke subtypes (e.g., cardioembolic) have an increased recurrence risk compared to others, suggesting that a tailored prevention program in this subgroup may be advisable (4). Recurrent strokes are associated with a longer hospital stay, higher mortality, and a higher degree of disability (5).

Cryptogenic strokes are at high risk of recurrences (4). The actual incidence is debated, but many studies have reported that about a third of all strokes are without a clear aetiology after the standard diagnostic workup – including CT or MRI angiography, echocardiography, and prolonged ECG-monitoring (6–8). Among cryptogenic strokes, the term embolic stroke of undetermined source (ESUS) indicates patients with non-lacunar strokes in which the most common cardioembolic sources have been excluded. ESUS accounts for about 17% of all strokes and is associated with a significant recurrence rate despite adequate antiplatelet treatment (9).

Accordingly, specific management for cryptogenic strokes still represent a debated field in clinical practice (10). New tools as smartphones and smart devices are becoming increasingly available and can record different types of health data such as heart rate (HR) and motion but also electrocardiogram (ECG), blood pressure (BP) or peripheral oxygen saturation (11). This represents a unique chance to monitor patients’ health for a prolonged period outside the hospital setting, especially for those affected by chronic diseases.

The present study aims at testing the feasibility of a smart device based secondary prevention program in a cohort of patients with cryptogenic stroke. We evaluated the compliance to the use of smart devices - capable of recording different types of health data - in a real-life scenario. Accordingly, we assessed the number of measures performed during the observation period.

## Methods

The study population included patients with non-disabling ischemic stroke – defined as a National Institutes of Health Stroke Scale (NIHSS) score < 3 (12) – or transient ischemic attack (TIA) in which the usual diagnostic workup – i.e. CT or MRI angiography of carotid arteries and intracranial vessels, 24-h ECG-monitoring, echocardiography – had not revealed any pathological alteration. This study was conducted accordingly to the declaration of Helsinki and was approved by the Ethics Committee of Università Campus Bio-Medico di Roma (Rome, Italy). Each participant gave a written informed consent before entering the study.

**Figure 1:**
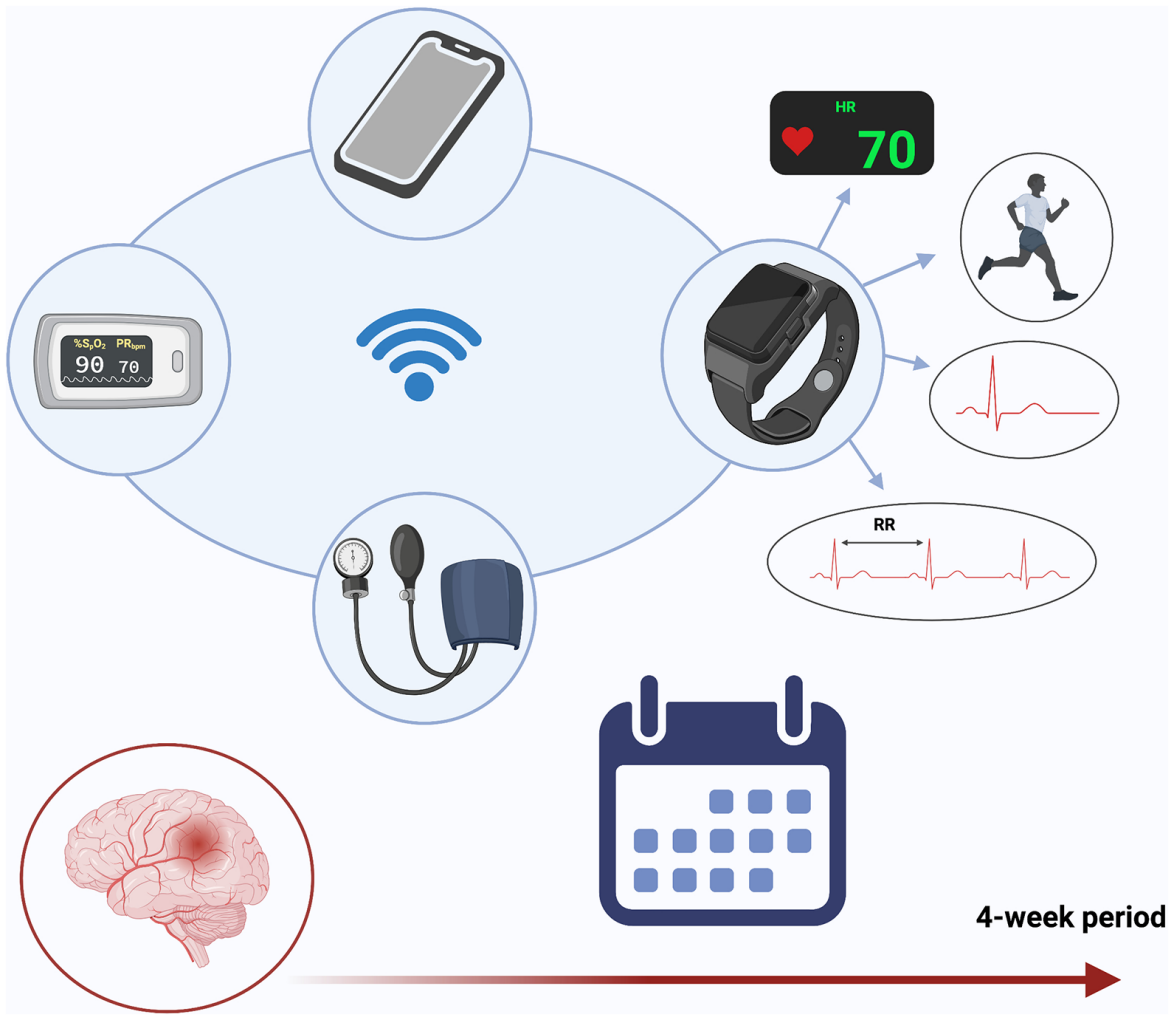
WATCH-group study kit. Patients enrolled in the WATCH group received a smartwatch – capable of recording heart rate, heart rate variability, footsteps, and ECG (the latter requires active participation of patients) – and a wireless blood pressure monitor and pulsi-oximeter. Data were individually collected on a smartphone that was set up on the day of the enrolment. Patients also received instructions on how to use the devices, both orally and in written form. The study ended after 4 weeks (created with BioRender.com).

Patients were recruited consecutively from our neurology department in the period from September 2020 to April 2021. We gathered the following baseline data: age, sex, education, complete medical history – including family history, previous cardiovascular events, and risk factors assessment - and the NIHSS or ABCD^2^ score - a risk assessment tool to predict the risk of stroke after a TIA - upon patient arrival at the emergency department. A history of atrial fibrillation (AF) or cognitive impairment as indicated by a Mini-Mental State Examination (MMSE) < 27 were exclusion criteria. All patients were on antiplatelet therapy at the enrolment, presented a similar risk of stroke recurrence – as ascertained through the Essen Score (13), a clinical score to identify patients at highest risk of recurrences in the year after a TIA/stroke - and received education about the secondary prevention of cerebrovascular disease. Each patient underwent a complete blood examination – including serum lipids, liver and kidney function, and thyroid hormones. Participants were then assigned to the smartwatch-based prevention program– i.e. WATCH group – or to the standard of care group – i.e. CONTROL group, according to a random matrix created on Matlab (The MathWorks Inc., Massachusetts, United States, Version: R2020a). As routine practice, all patients were scheduled with a follow-up visit roughly one-month after the acute event. Besides, patients of the WATCH group received a smartwatch – i.e. Apple Watch Series 4 (Apple Inc., Cupertino, United States) – capable of reliably detecting AF through a single-lead ECG (14). They also received a Bluetooth blood pressure monitor (iHealth BP7 wireless wrist BP monitor, iHealth Labs Inc., San Francisco, United States), a pulse oximeter (iHealth Air, iHealth Labs Inc., San Francisco, United States) and a smartphone – i.e. Apple iPhone series 6 (Apple Inc., Cupertino, United States) – to store the data collected during the 4-week observation period. Patients of the WATCH group were instructed to perform ECGs and to measure blood pressure and oxygen saturation at least once daily and especially in case of symptoms like shortness of breath or palpitations (Figure 1). No specific instructions were provided to patients in the CONTROL group, except for the usual education regarding stroke secondary prevention.

### Smart devices

The Smartwatch used in the study was equipped with various sensors. The main two of them were the enhanced PhotoPlethysmoGram (PPG) and the “heart sensor” to record ECG. The former contains a light-emitting diode (LED) that illuminates the skin and a diode measuring changes in light absorption caused by blood flow, thus recording the heartbeat rate (HR) and heartbeat variability – i.e., the interval between heartbeats in milliseconds (ms). The Apple Watch Series 4 was the first mass-produced consumer accessory receiving FDA approval for its Electrocardiogram (ECG) (15). The heart sensor comprises two electrodes integrated on the backside of the watch and a third one in the digital crown. This allows customers to take a single-lead ECG from their wrist, just by holding their index finger on the digital crown for an adequate amount of time (i.e., 30 s). Then the recording is classified as atrial fibrillation, sinus rhythm, low or high HR, inconclusive, or poor quality by the embedded ECG app, which accordingly sends a notification to the user. A PDF of the recording is also stored on the smartphone app “Health” (Apple Inc., Cupertino, United States). The smartwatch also included other sensors like the gyroscope, the accelerometer – that records arm motion – and a global position system (GPS) tracker, allowing accurate footsteps count.

The blood pressure monitor and the pulse oximeter worked through the app “iHealth My Vitals” (iHealth Labs, Inc., Mountain View, United States). The latter allows the customers to navigate among the available devices and measure the BP or Oxygen saturation, providing visual instruction on how to use the devices. These measures are automatically stored on the “Health” app of the smartphone.

At enrolment, patients in the WATCH group received the devices and the smartphone to store the data. This kit was controlled remotely through a mobile device management – ZuluDesk (Jamf, Minneapolis, United States) – to supervise devices, lock in the app store and limit internet access. However, personal data stored in the “Health” app on the smartphone were not accessible remotely, thus all the measurements performed by the patient were reviewed offline at the end of the study.

During enrolment, patients and caretakers received a 30-min training on how to use devices and solve the most common technical issues. They also received a brief instructions booklet and an email address to contact if there were any concerns.

Patients returned the devices at the end of the study period – i.e., after 4 weeks. Anonymized data were then individually exported from the “Health” app as .eXtensible Markup Language (.xml) files and then converted to comma-separated values (.csv) files. Using a custom-made semiautomatic script for Matlab (The MathWorks Inc., Massachusetts, United States, Version: R2020a), data were pre-processed and parameters of interest – that include HR, heart rate variability (HRV), step count, BP values, Oxygen saturation – were analyzed. Values of oxygen saturation below 85% in the absence of symptoms were considered as artefactual. Regarding ECG, the app “Health” stored all the data and PDFs of the ECG taken by the patients. The ECGs labelled as “Atrial Fibrillation” were then visually reviewed by one of the authors (F.M., A.M., C.V) to confirm the diagnosis.

### Outcomes

Our aim was to test the feasibility of a smart device based approach for stroke secondary prevention. Specifically, our primary endpoint was the number of times patients in the WATCH group adhere to our prevention program.

### Statistical analysis

All statistical analyses were carried out using the Statistical Package for Social Science (SPSS) program, version 25.0 (IBMCo., Armonk, NY) and Matlab (The MathWorks Inc., Massachusetts, United States, Version: R2020a). Data were expressed as mean or median [±SD and quartile ranges respectively] for continuous variables or as frequencies (n, %) for categorical data. Although papers about the use of smart devices as monitoring tools for patients with chronic conditions have already been published (15–17), owing to the proof-of-principle nature of the present study, no formal sample size calculation was performed. However, in previous studies with a similar design, sample sizes around 30 or greater have been recommended (18). Thus, we hypothesized that 200 subjects – grouped into the two arms – could be sufficient to test the feasibility of this approach. The Mann–Whitney rank-sum test and the *χ*^2^ test or Fisher’s exact test were used to compare variables. Statistical significance was defined as value of *p* < 0.05.

## Results

A total of 210 patients resulted to be eligible for this study. Thirty-four were excluded due to prespecified exclusion criteria - i.e., twenty-one patients presented an AF episode during hospital stay, thirteen did not meet the MMSE cut-off. Besides, fifteen patients withdrew from the study (twelve in the control group and three in the WATCH group). Data from one-hundred sixty-one patients were finally analysed of which eighty-seven patients were in the WATCH group and seventy-four in the standard group. One-hundred eleven had non-disabling strokes (69%) and fifty were TIA (31%). The main demographic and clinical characteristics are summoned up in Table 1.

**Table 1:** Baseline characteristics of the study population.

	Watch group (*n* = 87)	Standard group (*n* = 74)	*p* value
Age, median (Q1–Q3), y	70 (70–76)	70 (66–77.5)	*p* = 0.352
Women, *n* (%)	45 (51.7)	39 (52.7)	*p* = 0.903
Education			*p* = 0.577
Higher education, *n* (%)	27 (31)	20 (27)	
Secondary or lower, *n* (%)	60 (69)	54 (73)	
Stroke, N (%)	63 (72.4)	48 (64.9)	
NIHSS, median (Q1–Q3)	1 (1–3)	2 (1–3)	*p* = 0.087
TIA, *N* (%)	24 (27.6)	26 (35.1)	
ABCD^2^ score, median (Q1–Q3)	4 (3–5)	0 (0–2)	*p* < 0.001
Essen score, median (Q1–Q3)	2.5 (1–4)	3 (2–4)	*p* = 0.130
Arterial hypertension, *N* (%)	51 (58.6)	40 (54.1)	*p* = 0.563
Diabetes, *N* (%)	12 (16.2)	19 (21.8)	*p* = 0.370
Lipids			
LDL (mg/dl), mean (SD)	119.4 (39.2)	112.4 (36.6)	*p* = 0.308
Triglycerides (mg/dl), mean (SD)	130.8 (73.7)	114.9 (50.4)	*p* = 0.301
Smokers, *N* (%)	19 (21,8)	24 (32.4)	*p* = 0.246
Previous smokers, *N* (%)	20 (23)	18 (24.3)	
OSAS, *N* (%)	12 (13.8)	17 (23)	
BMI, mean (SD)	26.2 (4.4)	26.2 (5.6)	*p* = 0.617
Prior cerebrovascular disease, *N* (%)	17 (19.5)	13 (17,6)	*p* = 0.847
Family history of cardio- or cerebrovascular disease, *N* (%)	42 (48.3)	39 (52.7)	*p* = 0.308

Q1: first quartile; Q3: third quartile; SD: standard deviation; NIHSS: National Institutes of Health Stroke Scale; TIA: transient ischemic attack; LDL: low-density lipoprotein; OSAS: obstructive sleep apnea syndrome; BMI: body mass index.

The median NIHSS was 1 (Q1-Q3: 1–3) for patients in the WATCH group and 2 (Q1-Q3: 1–3) for patients in the control group. The two groups were similar in terms of Essen Score – median score WATCH group 2.5 (Q1-Q3: 1–4) versus 3 (Q1-Q3: 2–4). Subjects of both groups shared the same cardiovascular risk profile (i.e., smoking, lipid profile, hypertension, diabetes, Obstructive sleep apnea syndrome (OSAS), body mass index, history of cardiovascular disease) and demographic characteristics (age and gender). Regarding the latter, in both group the median age was 70 years. In the WATCH group there were 45 women (51.7%) while in the control group there were 39 women (52.7%).

**Figure 2:**
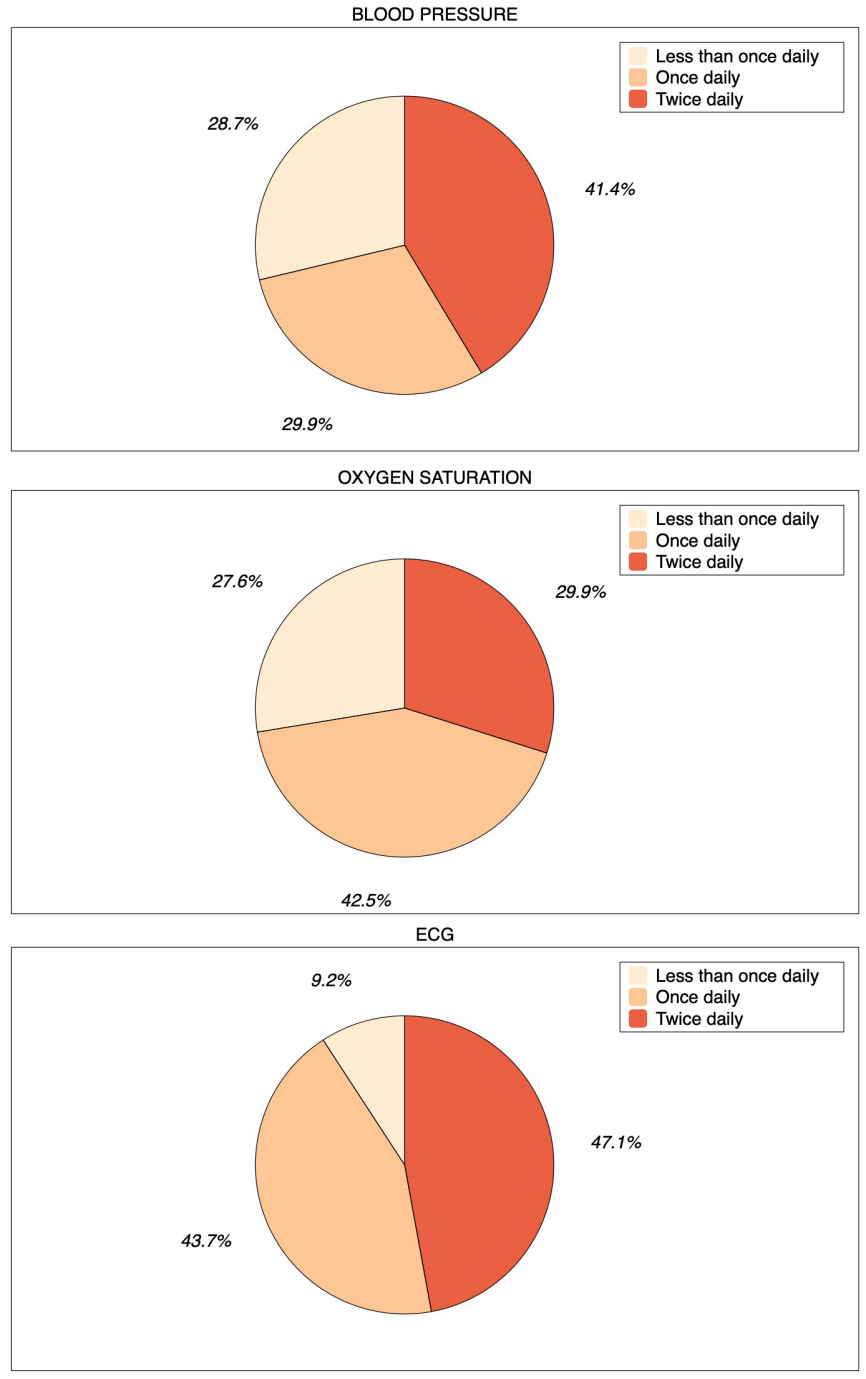
Pie charts depicting compliance during the study. Patients of the WATCH group were asked to record ECG and measure blood pressure and oxygen saturation multiple times daily, especially whenever they did not feel well. If not able to perform any measurement or ECG recording, they were considered as “non-compliant.”

**Figure 3:**
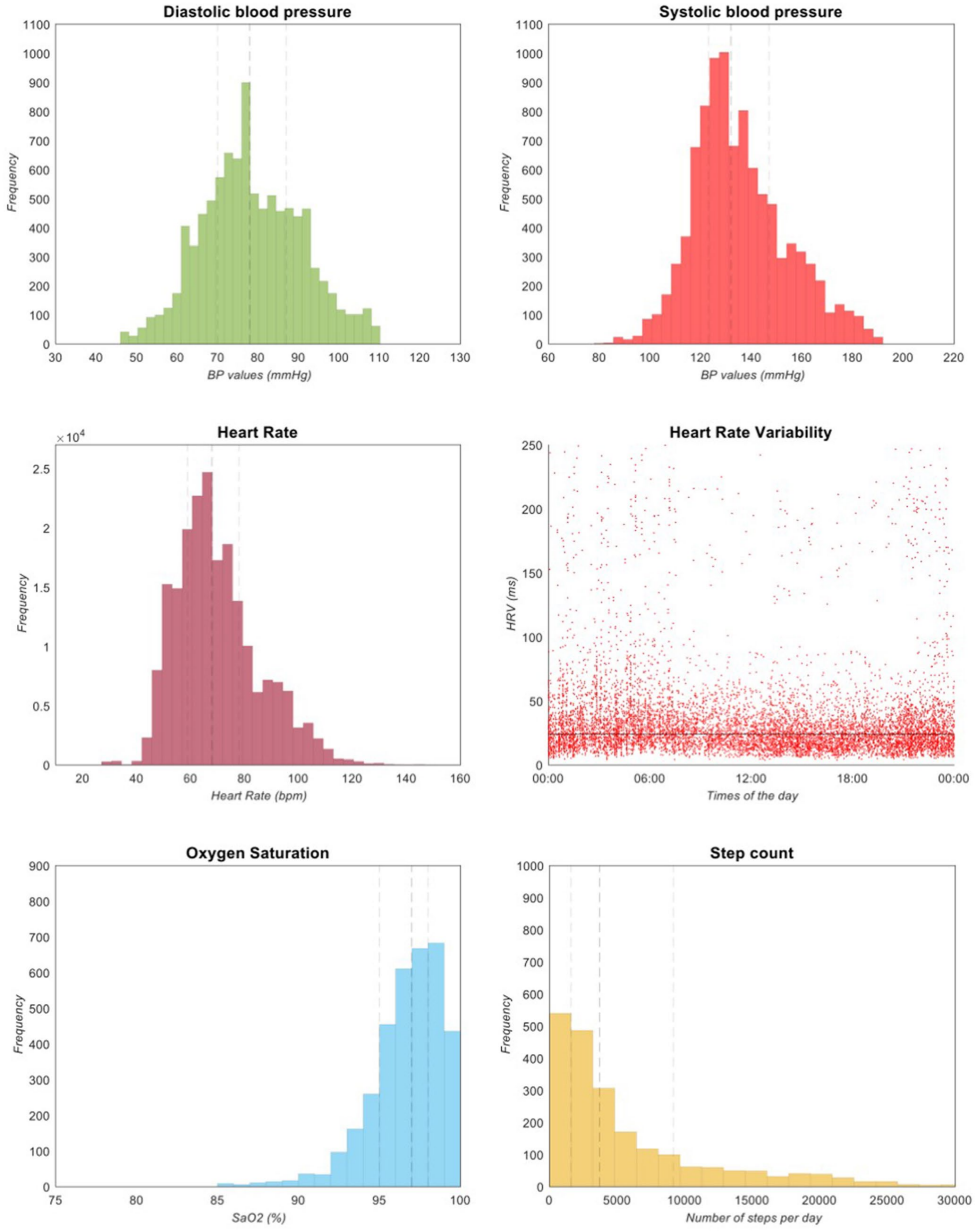
Parameters recorded by the devices in the WATCH group. Frequency distribution of BP values, Saturation values and step count. A scatter plot of HRV in function of time of the day is also plotted. The dashed lines represent the median (black), the Q1 and Q3 (grey) values. Q1: first quartile; Q3: third quartile; BP: blood pressure; HRV: heart rate variability; SaO2: oxygen saturation.

In the WATCH group, sixty-three patients (90.8%) recorded the ECG at least once a day. Forty-one patients (47.1%) of the entire cohort recorded the ECG twice a day (Figure 2). Eight patients (9.2%) were not able to record ECGs. In total, 5,335 ECGs – including inconclusive recordings (n = 1,650, 30.9%), i.e., of low-quality or too short to be analyzed by the algorithm - were recorded during the study. On average, each patient recorded about 61 ECGs during the 4-week study period. Thirty-six (41.4%) and twenty-six (29.9) patients measured blood pressure and oxygen saturation twice a day, respectively (Figure 2). The median systolic and diastolic blood pressure was 132 (Q1-Q3: 123–147) and 78 (Q1-Q3: 70–87) mmHg, respectively. During the study, 9,822 blood pressure measurements were attempted, of which 9,558 (97.3%) were valid. Oxygen saturation was measured 4,089 times and 3,499 (88.2%) measures resulted valid. Eighty-five measures (2.4%) resulted to be below 85% and were removed from the final analysis since they were isolated measures and were not associated to clinical symptoms. The median oxygen saturation value was 97 (Q1-Q3: 95–98) %. The median number of steps per day was 3,719 (Q1-Q3: 1663–9,154) as recorded by the smartwatch or the smartphone. During the study period, the smartwatch recorded 207,004 HR measures. The median heart rate was 68 (Q1-Q3: 59–78) bpm, while the minimum and maximum values recorded were 28 and 195 bpm, respectively. The smartwatch also measured HRV by monitoring the time interval between two consecutive heartbeats. In particular, the median value of the HRV was 24.29 (Q1-Q3: 16.73–36.41) ms. These parameters recorded during the study are summarized in Table 2 and Figure 3. No issues or side effects were reported during the observation period.

**Table 2:** Values of the parameters recorded by the devices in the WATCH group.

	MEDIAN	Q1	Q3	MEAN	SD
Diastolic BP (mmHg)	78	70	87	78.43	12.36
Systolic BP (mmHg)	132	123	147	135.56	18.59
Heart Rate (bpm)	68	59	78	70.04	15.62
Heart Rate Variability (ms)	24.29	16.73	36.41	33.12	16.73
Oxygen Saturation (%)	97	95	98	96.18	2.28
Nr. of Steps per day	3,719	1,663	9,154	8,073	11,489

BP: blood pressure; Q1: first quartile; Q3: third quartile.

From a clinical standpoint, in the WATCH group, one patient (1.1%) had a recurrent cerebrovascular event – i.e., a minor stroke - and nine patients (10.3%) had at least one episode of AF detected by the smartwatch app. In the CONTROL group, three patients (4%) were diagnosed with AF during the observation period. These diagnoses were done independently from the investigators of the present study. Besides, no cases of recurrent stroke were reported in this group. There was no statistically significant difference between groups for episodes of AF (*p* = 0.13) or cerebrovascular events (*p* = 0.355).

## Discussion

The potential for improving the management of neurological disorders of smart health devices is well-demonstrated by a vast body of literature (11). Since the number of smart device owners is growing worldwide, this will likely change the doctor-patient relationship. Smart devices are embedded with sensors that record many different parameters – like continuous motion data, HR and HRV (11) and also ECG (19) - and numerous studies have shown that they may be helpful for monitoring functional status in Multiple Sclerosis (20), for improving compliance and outcomes of rehabilitation in chronic stroke survivors (21), for providing personalized support to people affected by dementia (22), for a better characterization of tremor in Parkinson’s disease and essential tremor (23, 24), just to mention a few. However, to the best of our knowledge, this is the first study in which an integrated smart devices bundle has been used to monitor stroke patients in the subacute phase, when the recurrence risk is higher. Even if there are plentiful data regarding the potential application of wearables in medicine, in this study we investigated a novel approach for secondary prevention in stroke survivors. Our results suggest that this approach might be implemented even with the current digital literacy of the general population and might be helpful for the subgroup of stroke patients at highest risk of recurrences to extend the monitoring in a non-invasive manner. This approach is extremely cost-effective and, in our opinion, could represent an additional valuable resource for monitoring patients after stroke. Besides, the compliance to our program and the overall quality of the data recorded were satisfactory, especially considering the heterogenous population.

Evidence suggests that stroke incidence might be reduced by 50% through an adequate prevention program including the comprehensive management of cardiovascular risk factors, such as hypertension, diabetes, obesity, physical inactivity, and heart disease (1). Considering the dramatic impact of stroke from an epidemiological standpoint, there is a clear need of improving prevention, especially for patients with mild strokes or TIA.

In this regard, AF is the most common disorder of heart rhythm and affects about 40 million people worldwide (25). It is associated with a 5-fold risk of stroke (26) and is estimated to be the cause or a contributing factor in 15–20% of strokes (27). Two recent clinical trials aimed at investigating the role of covert AF in ESUS patients failed to demonstrate a clinically significant advantage of oral anticoagulation in this population, even if data suggests an indication for longer ECG monitoring in specific patients’ subgroups - i.e. older than 75 years when AF is actually more common (28). However, prolonged monitoring may be time- and resource-consuming and, even if current stroke guidelines include a recommendation for post-discharge heart-rhythm monitoring (29), there is no indication about how long this should be extended. In the absence of definite guidelines or reliable best practices, there is a clear need for innovative solutions to help clinicians tailoring prevention programs on patients’ characteristics. In this regard, ECG-recording smart devices offer the chance to augment our ability to detect arrhythmias in a real-life scenario (14).

In our cohort, although not statistically significant, the number of AF episodes was higher in the intervention group (10.3 *vs* 4%), despite the short duration of the observation period. Smart devices are capable of detecting AF episodes, being almost as reliable as conventional tools (30), and they may also be used by the more vulnerable groups of population – such as the elderly – owing to their user-friendliness. However, smartwatches provide only a single-lead ECG, and no information about AF episode duration is available. The latter may be critical for the eventual decision of starting anticoagulation since many authors have questioned the actual role of short duration AF episodes in stroke pathogenesis (31). Another frequent criticism is that older people or people with a lower level of instruction might have difficulties using these technologies. In our study, the vast majority of patients took at least one ECG per day (>90%) and more than two-thirds of the recorded ECGs were of sufficient quality to be analyzed by the in-built smartwatch algorithm. Considering the variegate nature of our cohort in terms of age and instruction, it is reasonable to say that the current intellectual resources are enough for implementing new technologies in clinical practice, as also shown by other similar experiences (32, 33). Indeed, the recent Coronavirus Disease 2019 pandemic had the unexpected twist of prompting the implementation of remote monitoring and telemedicine programs around the world and the number of papers on this topic has skyrocketed in the last 2 years (34).

At present, smartwatch customers can take an ECG at discrete times and with active engagement but, through the application of machine learning algorithms, it is also conceivable the passive detection of AF using smartwatch data (35). This will make the implementation of health gadgets even easier in daily life. Other measures can be also valuable, such as HRV, corresponding to the physiological variation of the time interval between consecutive heartbeats. HRV is an index of autonomic nervous system functioning and is considered normal between 20 and 200 ms. Some authors have suggested that increased HRV may predict the onset of AF, indicating an imbalance in the cardiac vagal tone (36). HRV is one of the most promising biomarkers of AF and is recorded passively, thus its use in daily life scenarios deserves more research (Figure 3).

Even if detecting AF is the most common outcome in many studies regarding wearables, the other parameters recorded by the smart devices might be extremely important for tailoring prevention program. In this study we investigated HR, BP, SaO2 and step count. These are critical for the management of other types of strokes, such as lacunar or atherothrombotic strokes.

The role of these measures is more evident at the individual rather than group level. For instance, patients who showed HR < 40 bpm on multiple occasions were scheduled with a cardiologic evaluation. Whilst patients with lower levels of physical activity – the median value of daily footsteps was disappointingly low in our study, i.e., ~3,700 steps/day - were advised to increase the time dedicated to exercise, highlighting the long-term benefit on cardiovascular fitness (37). We have to acknowledge that patients have reported some technical issues using the BP and SaO_2_ monitors. These were third-part devices managed by a specifically designed app and sometimes users experienced compatibility issues, e.g., Bluetooth pairing not working on several occasions. This was also the reason for the lower compliance we noted for BP and SaO2 measures. Integrated environments make the user experience smoother and might stimulate patients’ engagement, thus improving compliance. In our opinion, the next step is to develop an integrated app capable of managing different devices and offering a better and simpler user experience. This specifically designed app should also provide an online connection with clinicians that might be informed in real-time of red flags, i.e., very low-HR or elevated BP. Alongside this real-time warning system, the other parameters – HRV or step count – may be reviewed at different timescales to have the overall vision of how the patient is doing. One could also imagine specific training programs to increase compliance for sedentary patients that do not accomplish the required exercise targets. Or, in general, specific tips on how to improve lifestyle.

Remote monitoring programs – like the one we set up for this experiment – might represent the opportunity to actively engage patients in the therapeutic relationship. Indeed, despite our concerns - Italy is one of the European countries with the highest digital illiteracy (38) - patients adherence to the study protocol was overall satisfying since 90% of patients recorded the ECGs at least once a day and the quality of data of the other parameters – such as BP, SaO2 and steps – was satisfactory.

Another issue to deal with is the vast amount of data that can be recorded. At present, only a small fraction of it might have a substantial role in helping clinicians make informed clinical decisions. Handling big data is a great challenge for healthcare providers. Biometric sensors from wearables record a large volume of unstructured data that must be processed into meaningful structured information, keeping in mind the “Mantra” of big data analytics, i.e., the more the data, the more insight one can gain from them. This is necessary for improving our skills in managing acute conditions (39) and making clinical predictions, providing early warnings of disease progression, finding novel biomarkers, just to mention a few (40). Thus, it is mandatory to carefully choose the quality and quantity of data to provide to clinicians, without neglecting yet another issue, i.e., the importance of protecting our patients’ data. Indeed, the safety of personal data is of paramount importance for the definitive implementation of new technologies in daily routine, but it is an often-overlooked concern. There is still a long road ahead before the definitive implementation of these technologies in clinical practice. However, investing in technological ecosystems to improve patients’ user experience and quality of recorded data would pay substantial returns in terms of better management of diseases and the reduction of the socioeconomic burden of healthcare costs on society.

This study has some limitations to be addressed. Firstly, the limited sample size was defined to verify the feasibility of our approach and not to investigate differences between the two groups. Secondly, the short duration of the study that was chosen to investigate the stroke subacute phase, in which the risk of recurrence is higher. However, it is reasonable to say that longer observation periods might offer a better chance of finding abnormalities. For instance, it is not clear what is the optimal time window for ECG monitoring and, in our opinion, more extended observation periods may be useful due to smartwatch cost-effectiveness. Then, we have to acknowledge that some measurements could be inaccurate due to incorrect use of devices. This especially applies to the blood pressure monitor and pulse oximeter. Also, we recorded patients’ satisfaction only informally, without implementing any specific questionnaire. In an eventual follow-up study, it would be interesting to look at patients’ satisfaction for this kind of approach. Another limitation is the lack of data about sleep. After reviewing some of the most common apps for the iOS environment, we decided not to include another third-part app to our bundle to make the user-experience easier. Finally, we have to acknowledge the slight heterogeneity of the groups, which was probably due to the “real-life” study design, i.e., patients were recruited consecutively from our Clinic according to inclusion and exclusion criteria and then enrolled. In our opinion, this latter point is also a strength of our study since it demonstrated that using new technologies is possible even with the current resources and the current digital literacy level of patients.

## Conclusion

Our study suggests that prevention programs for cerebrovascular disease may benefit from the implementation of new technologies. Collecting real-life data may be helpful to identify red flags in patients at high risk of stroke, eventually prompting medical attention. The use of remote monitoring devices – allowing the prolonged monitoring of different vital parameters – might radically change the doctor–patient relationship and help the implementation of individually tailored prevention programs (11). In the next future, physicians could have access to a large amount of real-life health data regarding their patients and these data will be used for the prevention of acute diseases or for the monitoring of chronic conditions. Ideally, data will be transmitted to healthcare professionals in real-time, thus abating the latency of traditional in-person visits. However, more studies on a larger sample size are warranted to investigate the efficacy and cost-effectiveness of this approach versus the standard of care.

## Data availability statement

The raw data supporting the conclusions of this article will be made available by the authors, without undue reservation.

## Ethics statement

The studies involving human participants were reviewed and approved by The Ethics Committee of Università Campus Bio-Medico di Roma (Rome, Italy). The patients/participants provided their written informed consent to participate in this study.

## Author contributions

FM, FC, and VL conceived and designed the study. AM, CV, and GI recruited the patients and collected the data. FM, FC, EF, and FP analyzed the data and contributed to data interpretation. FM wrote the first draft of the manuscript. FC, FP, and VL reviewed the manuscript. All authors contributed to the article and approved the submitted version.

## Funding

This work was funded by the “Associazione Nazionale fra le imprese Assicuratrici” (ANIA).

## Conflict of interest

FC has received travel grants from Biogen, Merck, Teva and Sanofi-Genzyme.

The remaining authors declare that the research was conducted in the absence of any commercial or financial relationships that could be construed as a potential conflict of interest.

## Publisher’s note

All claims expressed in this article are solely those of the authors and do not necessarily represent those of their affiliated organizations, or those of the publisher, the editors and the reviewers. Any product that may be evaluated in this article, or claim that may be made by its manufacturer, is not guaranteed or endorsed by the publisher.
